# Two-step breakdown of a SiN membrane for nanopore fabrication: Formation of thin portion and penetration

**DOI:** 10.1038/s41598-018-28524-5

**Published:** 2018-07-04

**Authors:** Itaru Yanagi, Hirotaka Hamamura, Rena Akahori, Ken-ichi Takeda

**Affiliations:** Hitachi Ltd., Research & Development Group, Center for Technology Innovation - Healthcare, 1-280, Higashi-koigakubo, Kokubunji, Tokyo 185-8603 Japan

## Abstract

For the nanopore sensing of various large molecules, such as probe-labelled DNA and antigen-antibody complexes, the nanopore size has to be customized for each target molecule. The recently developed nanopore fabrication method utilizing dielectric breakdown of a membrane is simple and quite inexpensive, but it is somewhat unsuitable for the stable fabrication of a single large nanopore due to the risk of generating multiple nanopores. To overcome this bottleneck, we propose a new technique called “two-step breakdown” (TSB). In the first step of TSB, a local conductive thin portion (not a nanopore) is formed in the membrane by dielectric breakdown. In the second step, the created thin portion is penetrated by voltage pulses whose polarity is opposite to the polarity of the voltage used in the first step. By applying TSB to a 20-nm-thick SiN membrane, a single nanopore with a diameter of 21–26 nm could be fabricated with a high yield of 83%.

## Introduction

Recently, nanopore sensing has become a powerful method for directly probing biomolecules in aqueous solution. Ionic current through a nanopore changes while the target molecule passes through it, and this change in current can be used to determine the structural and electrical characteristics of the molecule. The most well-known application of nanopore sensing is label-free single-molecule DNA sequencing (i.e., nanopore DNA sequencing)^1–11^. According to the latest report^11^, the sequencing of a human genome was attained with high accuracy using biological nanopore DNA sequencers distributed by Oxford Nanopore Technologies, Ltd. Nanopores are also used as molecular-sized Coulter counters^12–20^. For example, the monitoring of single-molecule immunoreactions was demonstrated by counting the number of current-blockade events derived from antigen-antibody complexes passing through a nanopore^19^. In addition, target DNA sequence detection has also been demonstrated by binding peptide nucleic acid (PNA) or PNA-polyethylene glycol (PEG) labels to the target sequences and detecting the changes in current resulting from the labelled DNA passing through a nanopore^20^. These sensing techniques do not require optical measurements and have the potential to be used in portable diagnostic devices in the future.

Solid-state nanopores are suitable for such large-molecule detection applications, i.e., detecting molecules larger than DNA. Compared to biological nanopores, which are typically limited to 2 nm or less in size, solid-state nanopores are not limited in size and can be customized in accordance with the sizes of the target molecules. In fact, Morin *et al*. prepared solid-state nanopores with diameters of approximately 20–30 nm to detect PNA-PEG-labelled DNA^20^. In addition, for such applications, the nanopore thickness does not need to be ultrathin, as is required for DNA sequencing with single-nucleotide resolution. In fact, from a durability perspective, thicker nanopores would be preferable.

Several methods have been developed to form a nanopore in a solid-state membrane^21–31^. For instance, electron beam (EB) lithography and etching enables the fabrication of nanopores down to approximately 20–30 nm in diameter^22^. Even smaller nanopores down to sub-2 nm in diameter can be fabricated by focused-electron-beam etching^23,24^ or helium ion etching^25–27^ by transmission electron microscopy (TEM) or helium ion microscopy (HIM). Moreover, nanopore fabrication by utilizing dielectric breakdown of a membrane has also been demonstrated in many reports^28–46^. According to the first report^28^, by Kwok *et al*., a nanopore down to sub-2 nm in size could be created by applying a high constant voltage to a membrane via aqueous solutions and terminating the voltage when a current through the membrane reached a predetermined cut-off value. This method is called “controlled breakdown (CBD)”. Nanopore fabrication by dielectric breakdown has a great advantage in terms of fabrication cost; expensive equipment, such as that needed for EB lithography, TEM or HIM, is not required. However, this method is rather unsuitable for the stable fabrication of a single large nanopore due to the risk of generating multiple nanopores^33,34,36,37^ when trying to widen a nanopore by additional voltage stresses. Therefore, most of the nanopores used in previous studies^29–31,40–46^ fabricated by dielectric breakdown were less than approximately 5 nm in diameter.

In this study, the stable fabrication of a single large nanopore (larger than 20 nm in diameter) in a thick SiN membrane (20 nm in thickness) via dielectric breakdown is demonstrated. In the process of achieving this large nanopore fabrication, we found several new phenomena. First, a nanopore was not generated in the SiN membrane by CBD if the cut-off current was set below a certain value. Instead, a local thin portion was created in the membrane. As the cut-off current was increased, the thin portion was expanded and multiple nanopores were generated. The time from the formation of the thin portion to the generation of the multiple nanopores was only 0.2 s or less. Consequently, it was practically impossible to fabricate a single nanopore, regardless of its size.

To form a single nanopore in the SiN membrane, we propose a technique called “two-step breakdown” (TSB). In the first step of TSB, a local thin portion is created by CBD with a relatively low cut-off current. In the second step, a nanopore is fabricated by penetrating the thin portion with voltage pulses. The polarity of the voltage pulses is opposite to the polarity of the voltage during CBD in the first step. By using TSB, nanopores with effective diameters of 21–26 nm could be fabricated with a high yield of 83%.

## Results

**Figure 1:**
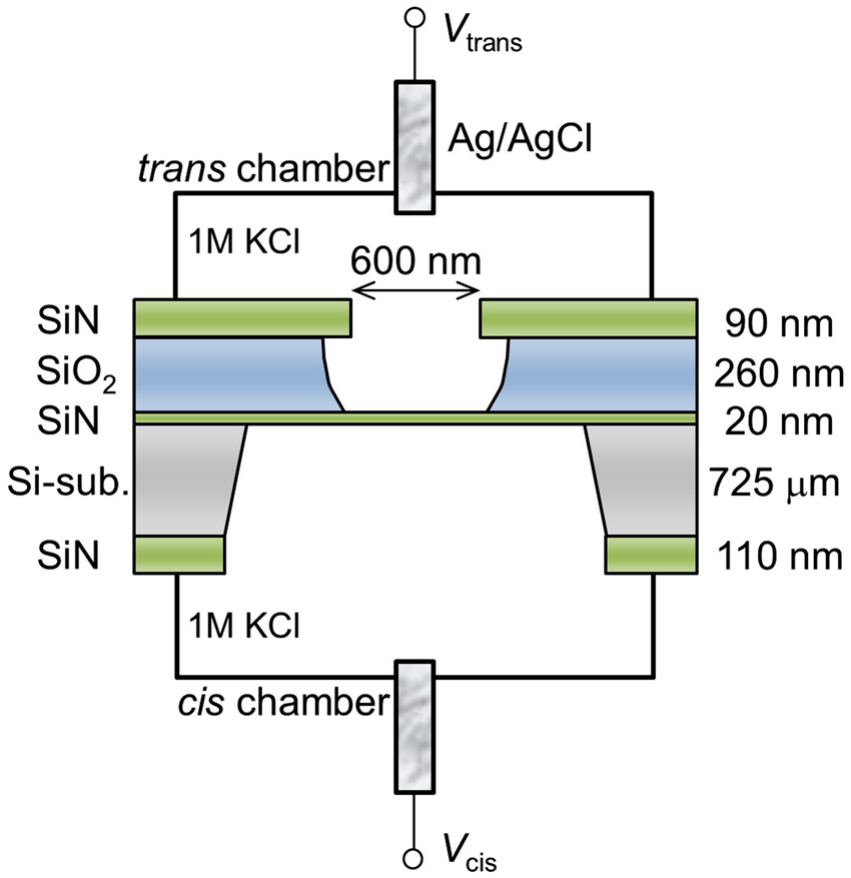
Schematic illustration of experimental setup. *Cis* and *trans* chambers are separated by a SiN membrane with a thickness of 20 nm. Both chambers were filled with 1 M KCl aqueous solution. Two Ag/AgCl electrodes were immersed in both chambers and connected to voltage source units and ammeters.

**Figure 2:**
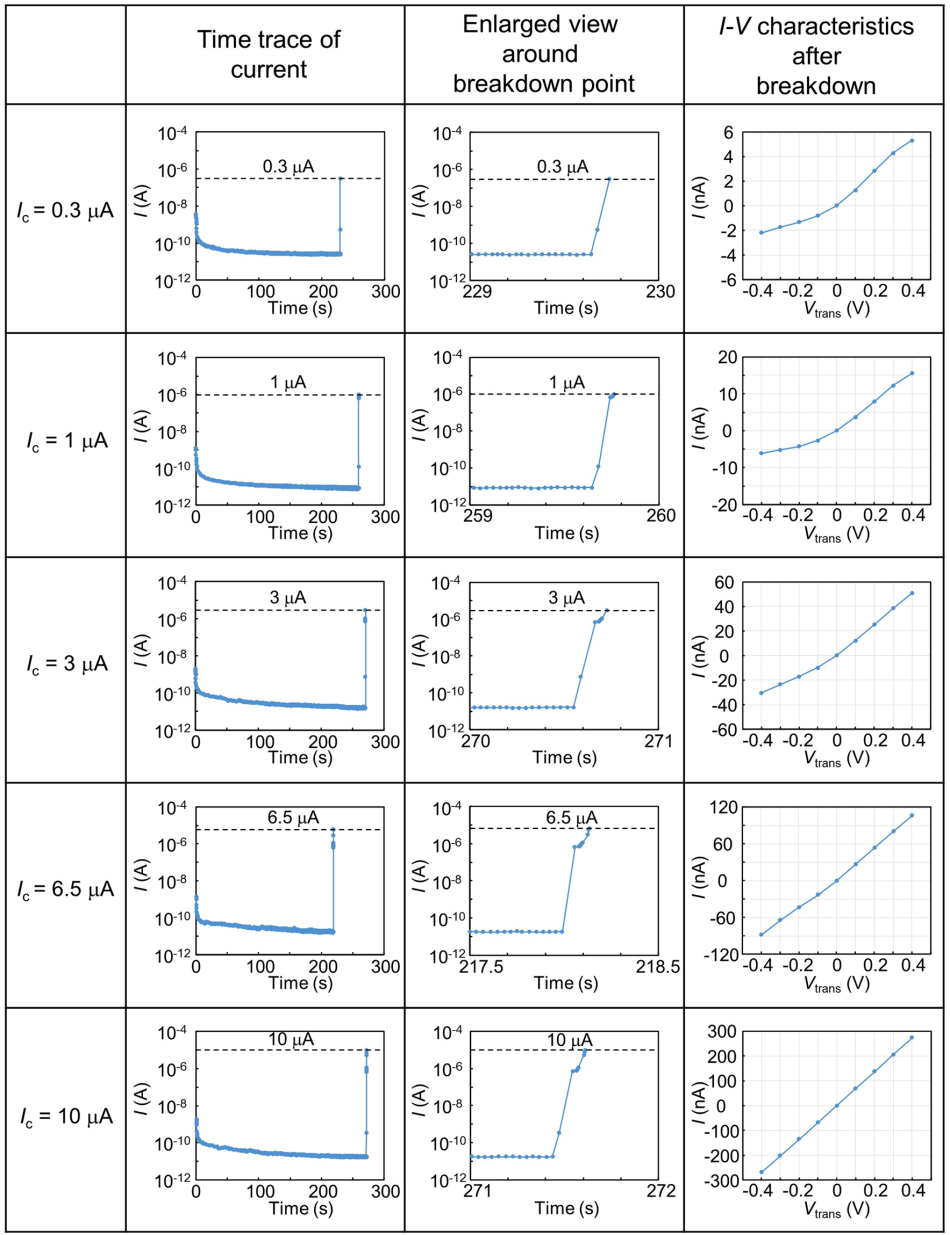
Dielectric breakdown of 20-nm-thick SiN membranes by CBD with different cut-off currents (*I*_c_). Graphs in the left column present time traces of currents through the membranes during CBD. Graphs in the middle column present magnified time traces around the dielectric-breakdown points. Graphs in the right column present *I-V* characteristics after CBD. *V*_cis_ was set at 0 V during the measurements of *I-V* characteristics.

The setup for the dielectric breakdown experiment is illustrated in Fig. 1. The area of the 20-nm-thick SiN membrane was restricted within a small square area (approximately 600 × 600 nm^2^) such that fabricated nanopores could be easily found. Cross-sectional TEM images of the SiN/SiO_2_/SiN multilayer film on the Si substrate are shown in Supplementary Fig. 1. Two Ag/AgCl electrodes (*cis* and *trans* electrodes) were immersed in 1 M KCl aqueous solution with pH = 7.5 for applying voltages and measuring currents through the membrane. In this study, *V*_cis_ was set at 20 V, and *V*_trans_ was set at 0 V for CBD. Figure 2 presents the time traces of currents during CBD, enlarged time traces around the dielectric-breakdown points, and *I*-*V* curves after CBD. Five representative characteristics with five different cut-off currents (*I*_c _= 0.3–10 μA) are presented. The dielectric-breakdown point was clearly observed in each time trace of the current. Each *I*_c_ was set as a current compliance value of the measuring instrument, i.e., *I*_c_ was a limit of the output current of the instrument, and the applied voltage automatically dropped when the current between the electrodes reached *I*_c _ ± 0.1%. Each enlarged time trace confirms each current reached each *I*_c_ less than 0.2 s after the occurrence of dielectric breakdown. The *I*-*V* characteristics after CBD changed from exhibiting an asymmetric behaviour to an ohmic behaviour as *I*_c_ increased.

**Figure 3:**
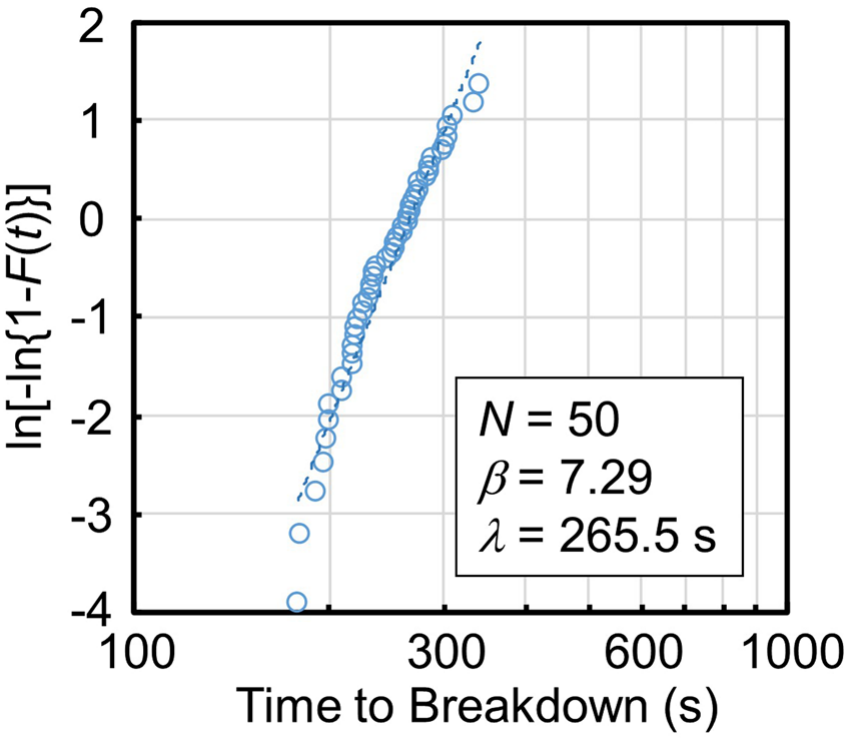
Weibull plot of the time-to-breakdown of the membranes. Fifty membranes were tested to investigate the time-to-breakdown statistics under the conditions of *V*_cis_ = 20 V and *V*_trans_ = 0 V. The dashed line represents a fitting line with equation (2). The fitting parameters are *β* = 7.29 and *λ* = 265.5.

Figure 3 presents a Weibull plot of the time to breakdown. The cumulative breakdown probability (*F*) by time *t* is defined as

1 $$F(t)=\frac{n(t)}{N},$$

where *N* = 50 is the total number of membranes tested, and *n*(*t*) is the number of membranes that experienced dielectric breakdown by time *t*. In a Weibull distribution derived from a weakest-link model^32,47–50^, *F*(*t*) is defined by the following equation:

2 $$F(t)=1-\exp (-{(\frac{t}{\lambda })}^{\beta }),$$

where *β* is the Weibull slope, and *λ* is the time at which approximately 63% of the membranes have experienced dielectric breakdown. The plots in Fig. 3 are well fitted by equation (2) with *β* = 7.29 and *λ* = 265.5 s, meaning that the breakdown events in our experiment can be explained by a weakest-link model, as reported in other previous studies^32,46^. Note that our SiN membranes have high immunity against dielectric breakdown. According to the results reported by Kwok *et al*.^28^ and Briggs *et al*.^32^, a 30-nm-thick SiN membrane in an aqueous solution of 1 M KCl at pH 7 is estimated to be broken by applying 18 V (i.e., 18 V/30 nm = 6 MV/cm in electric field strength) for a few hundreds of seconds. On the other hand, an application of 20 V for 200–300 s was needed to break down our 20-nm-thick SiN membranes (i.e., 20 V/20 nm = 10 MV/cm in electric field strength) under almost the same aqueous conditions. This difference is thought to be due to the differences in the basic material properties of the SiN membranes. The 30-nm-thick SiN membranes reported in Refs^28,32^ were low-stress (<250 MPa), silicon-rich membranes. In contrast, the SiN film employed in our experiment was a high-stress (approximately 1 GPa) film and not silicon-rich. NH_3_-rich conditions were used for the deposition of our SiN film (see “Methods” section), and the composition ratio of the SiN film analysed by X-ray photoelectron spectroscopy (XPS) was approximately Si:N = 1:1.24.

**Figure 4:**
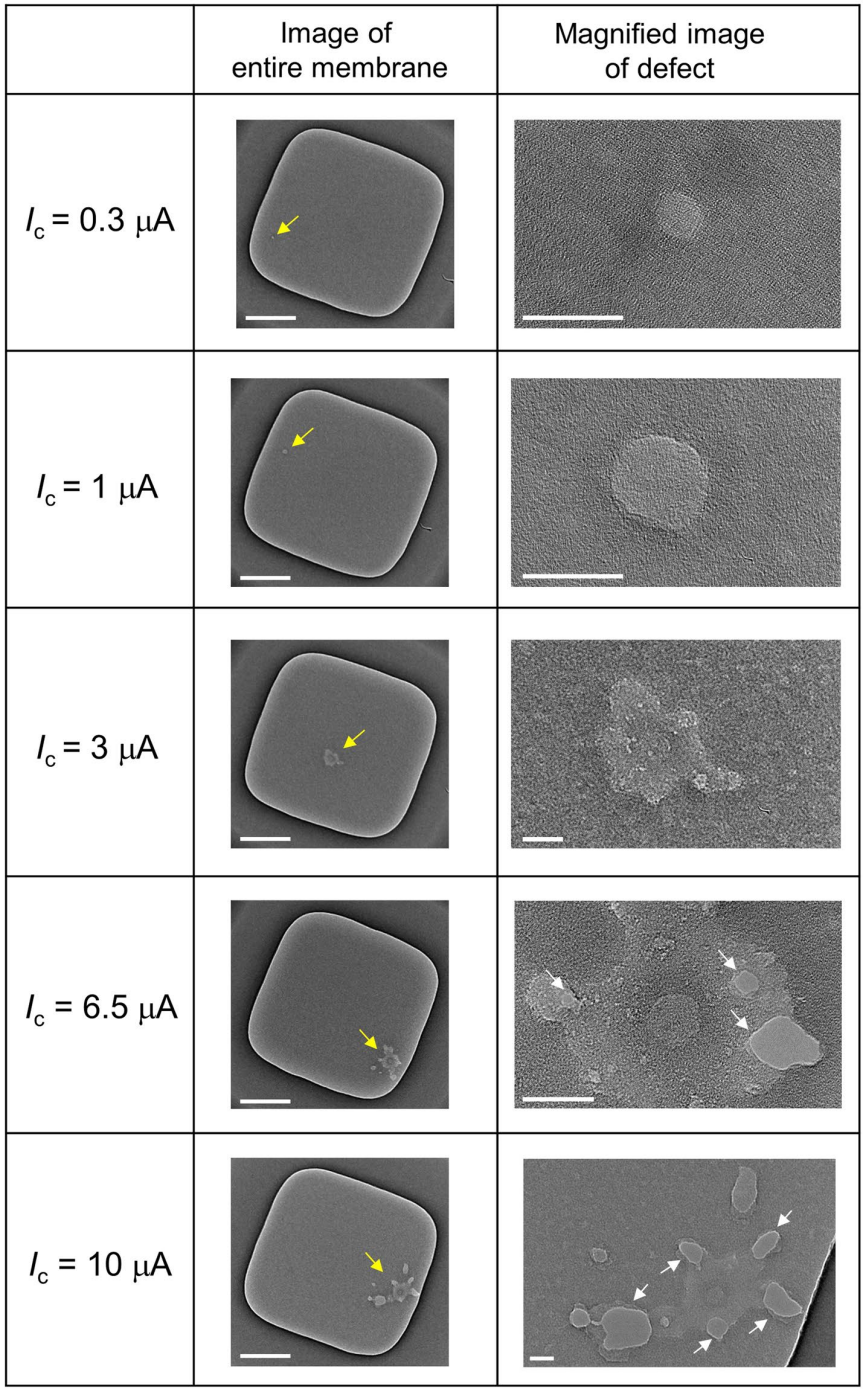
TEM images of the membranes after CBD with different cut-off currents (*I*_c_). Images of entire membranes are presented in the left column (scale bars are 200 nm). Defective portions are indicated by yellow arrows. Magnified images of the defective portions are presented in the right column (scale bars are 20 nm). Generated nanopores are indicated by white arrows.

Figure 4 presents TEM images of five different membranes after CBD at the five different *I*_c_ levels. Images of the entire membranes are shown in the left column. Defects created by CBD are indicated by yellow arrows. Magnified images of the created defects are shown in the right column. Interestingly, no nanopores were created in the membranes after CBD in cases of *I*_c_ ≤ 3 μA. Instead, a local thin portion was created in each membrane. From the magnified views, not nanopores but amorphous material is clearly confirmed in the defect areas. These areas are believed to have no electrical insulating property and allow charge translocations. We subjected 12 membranes to CBD with *I*_c_ ≤ 3 μA (i.e., 4 membranes for *I*_c_ = 0.3 μA, 5 membranes for *I*_c_ = 1 μA and 3 membranes for *I*_c_ = 3 μA), and no nanopores were confirmed in any of the membranes. Of course, as will be shown later, such thin portions did not allow the passage of DNA.

The thin area became larger as *I*_c_ increased, and multiple nanopores (indicated by white arrows) were generated in cases of *I*_c_ ≥ 6.5 μA. We subjected 6 membranes to CBD with *I*_c_ ≥ 6.5 μA (i.e., 4 membranes for *I*_c_ = 6.5 μA and 2 membranes for *I*_c_ = 10 μA), and multiple nanopores were confirmed in all of the membranes. Based on the results shown in Fig. 2, the time from the dielectric breakdown to the generation of multiple nanopores was less than 0.2 s. The generation of multiple nanopores within such a short time has not been reported and cannot be interpreted as an aggregation of independent weakest-link events. Theoretically, the probability wherein a breakdown event occurs *k* times in a membrane by time *t* is expressed by the Poisson distribution^48^:

3 $${P}_{k}(\mu (t))=\frac{{\mu }^{k}(t)\exp (\,-\,\mu (t))}{k!},$$

where *μ*(*t*) is the average number of dielectric-breakdown events by time *t*. Considering that *P*_0_ is the probability of no breakdown event occurring by time *t*, the following equation is obtained:

4 $${P}_{0}(\mu (t))=1-F(t)=\exp (-\mu (t)).$$

Consequently, *μ*(*t*) can be expressed as follows using equations (2) and (4):

5 $$\mu (t)={(\frac{t}{\lambda })}^{\beta }.$$

From equation (5), the average number of dielectric-breakdown events during 0.2 s (i.e., *μ*(*t* + 0.2 s) −*μ*(*t*)) is estimated to be less than 0.073 in the case of *t* = 100–400 s. Therefore, other explanations are required to interpret the generation of multiple nanopores. However, we cannot currently provide any credible explanations. One possible cause is the propagation of Joule heating energy via phonons in the SiN membrane. As described above, our SiN membranes required a higher voltage to be broken than that reported by others. As a result, a higher Joule heating energy (∝*I* × *V*) was generated after the breakdown, which might be high enough to create multiple nanopores in the membrane almost simultaneously. Another possible cause is change in the local stress of the membrane. Stress imbalances are generated near the boundary between the local thin portion and the other portion, which might cause ruptures in the membrane. In fact, most of the generated nanopores shown in Fig. 4 are located near the boundary of the local thin portion.

**Figure 5:**
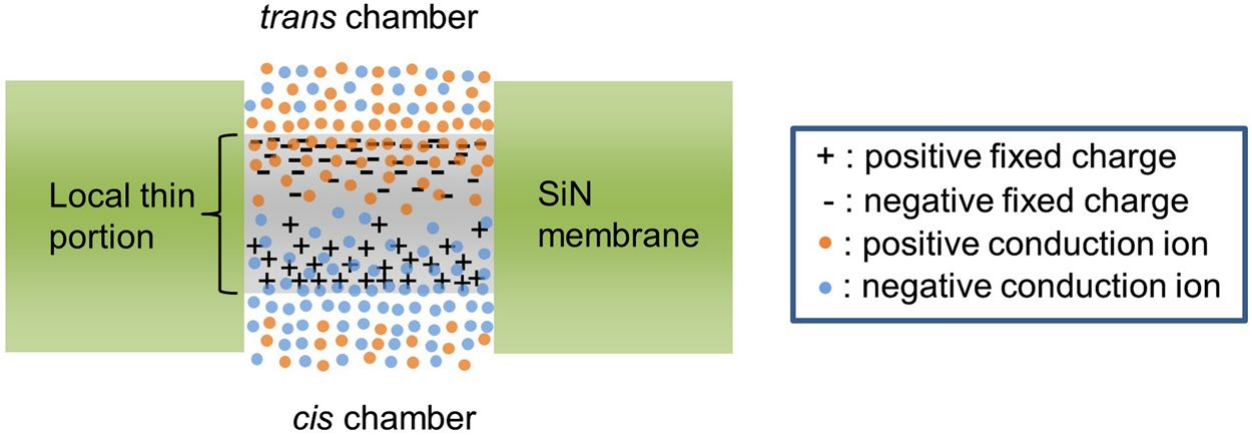
Schematic illustration of carrier distribution at equilibrium without a voltage bias after CBD. The + and − symbols represent positive and negative charges trapped in the local thin portion, respectively. Orange and blue dots represent positive and negative conduction ions, respectively.

Comparing the *I-V* characteristics in Fig. 2 and the TEM images in Fig. 4, significant rectifications are confirmed in the *I-V* curves before nanopore generation (i.e., for *I*_c_ ≤ 3 μA). These rectifications are attributed to the fixed charges, which were trapped in the local thin portion during CBD. Figure 5 illustrates a schematic of the carrier distribution in equilibrium without a voltage bias after CBD. The + and − symbols represent the positive and negative fixed charges (i.e., ions, electrons and holes in the trap sites), respectively. The orange and blue dots represent the positive and negative conduction ions, respectively. The fixed charges in the *cis* side are positive and those in the *trans* side are negative due to the voltage polarity (*V*_cis_ > *V*_trans_) during CBD. Then, the conduction ions distribute to compensate for the fixed charges. Such a PN-junction-like ion distribution has been reported in studies of nanofluidic channels^51,52^ and are known to exhibit diode characteristics. From the *I-V* curves shown in Fig. 2, *V*_trans_ > *V*_cis_ is a forward-biased state, and *V*_trans_ < *V*_cis_ is a reverse-biased state, which is consistent with those reports^51,52^. The diode characteristics become unclear and the ohmic response becomes dominant after the generation of nanopores.

Considering the above results, the stable fabrication of a single nanopore in our membrane is practically impossible by CBD with an applied voltage of 20 V in a KCl aqueous solution at pH = 7.5. Even if a single nanopore could be fabricated by tuning *I*_c_ to within 3 μA to 6.5 μA, it would be accompanied by a defective local thin portion. In fact, we observed two membranes which were subjected to CBD with *I*_c_ = 4.75 μA (see the TEM images in Supplementary Fig. 2). From the images, only local thin portions were confirmed (i.e., no nanopores were confirmed) in one membrane, whereas multiple nanopores were confirmed in the other membrane. This result indicates that the number of nanopores cannot be controlled by tuning *I*_c_. In addition, we observed membranes which were subjected to CBD under the conditions of pH 3 and pH 11 (see the TEM images in Supplementary Fig. 3). Only a local thin portion was formed in each of pH conditions when *I*_c_ = 1 μA, whereas multiple nanopores were generated in each of pH conditions when *I*_c_ = 6.5 μA. This tendency is the same as that in the case of pH 7.5. Of course, there is a possibility to find the pH conditions which enable the stable fabrication of a single nanopore if a wider range of pH is examined. Moreover, the voltage used in CBD is also a potential parameter which affects the defect forming process of dielectric breakdown. Detailed examinations of whether a single nanopore can be stably formed in the membrane by tuning these parameters will be addressed in future work.

**Figure 6:**
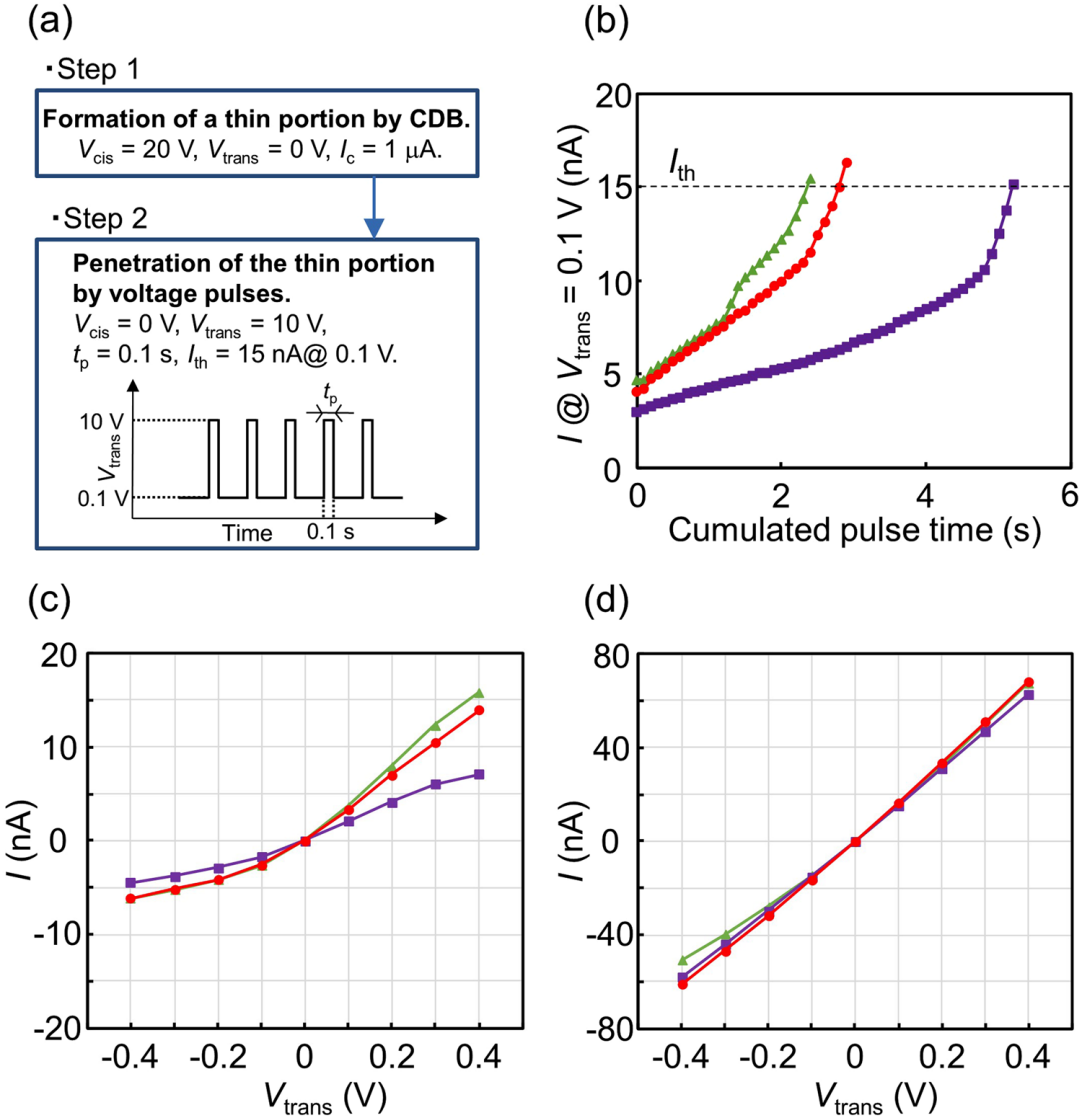
Electrical characteristics during TSB of the membrane. (**a**) TSB procedure. (**b**) Dependence of the current at *V*_trans_ = 0.1 V and *V*_cis_ = 0 V on the cumulated voltage-pulse time in the second step of TSB. (**c**) *I-V* characteristics across the membranes before the second step of TSB. (**d**) *I-V* characteristics across the membranes after the second step of TSB. The same-coloured plots in (**b**–**d**) are the results obtained from an identical membrane.

In this study, we propose a technique called “two-step breakdown” (TSB) to stably fabricate a single nanopore in the membrane. The TSB procedure is presented in Fig. 6(a). The first step is the formation of a local thin portion in the membrane by CBD. *V*_cis_ = 20 V, *V*_trans_ = 0 V and *I*_c_ = 1 μA were chosen to create a local thin portion with a diameter of approximately 20 nm, as shown in Fig. 4. The second step is the penetration of the created thin portion by voltage pulses (*V*_cis_ = 0 V, and *V*_trans_ = 10 V with a duration of 0.1 s). The polarity of the voltage pulses is opposite to the voltage polarity during CBD in the first step, which is important because a nanopore does not form in most cases when the same voltage polarity is used in the first and second steps (as shown in detail later). The current through the membrane at *V*_trans_ = 0.1 V was monitored after each voltage-pulse application. This iteration sequence was stopped when the monitored current at 0.1 V exceeded the threshold value (i.e., *I*_th_ = 15 nA). Figure 6(b) presents the dependence of the current at 0.1 V after each pulse on the cumulated pulse time. The results obtained from three different membranes are shown. Typically, the current exceeded *I*_th_ before the cumulated pulse time reached approximately 5 s. The *I-V* characteristics before and after the second step are presented in Fig. 6(c,d), respectively. The *I*-*V* characteristics became ohmic after the second step. Note that the same-coloured plots in Fig. 6(b–d) are the results obtained from an identical membrane.

**Figure 7:**
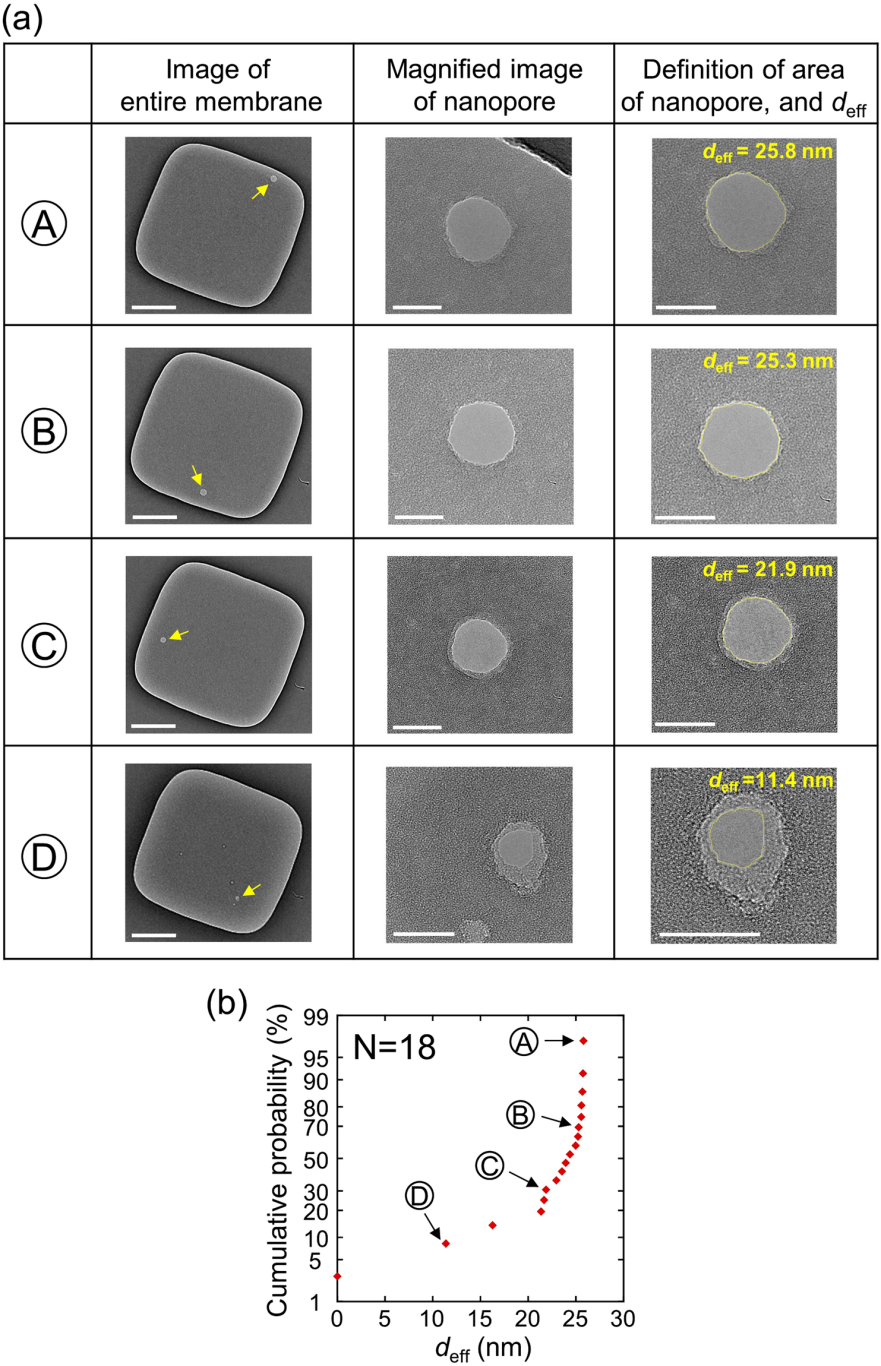
Nanopores fabricated by TSB when the polarity of the voltage pulses in the second step was opposite to that of the voltage in the first step. (**a**) Images of entire membranes are presented in the left column (scale bars are 200 nm). The portions of the fabricated nanopores are indicated by yellow arrows. Magnified images of the fabricated nanopores are presented in the middle column (scale bars are 20 nm). The areas of the nanopores surrounded by yellow lines and their effective diameters are presented in the right column (scale bars are 20 nm). (**b**) Cumulative probability of the effective diameters (*d*_eff_) of the fabricated nanopores.

TEM images of the fabricated nanopores by TSB are presented in Fig. 7(a), and the cumulative probability of the effective nanopore diameter (*d*_eff_) is presented in Fig. 7(b). The TSB conditions were set as described in Fig. 6(a). *d*_eff_ was defined as

6 $${d}_{{\rm{eff}}}=2{(\frac{S}{\pi })}^{1/2},$$

where *S* is the area of the nanopore surrounded by the yellow line, as shown in the right column of Fig. 7(a). The area was measured using image processing software (ImageJ). Compared to the TEM images after the first step, no amorphous material was confirmed in the hole region. In addition, the number of nanopores was one per membrane. (The portion of the nanopore is indicated by a yellow arrow in each image of an entire membrane.) Although several defective portions other than the nanopore portion were confirmed in the image of the entire membrane in Ⓓ, such defective portions were not nanopores but local thin portions. However, current could flow through the local thin portions. As a result, the fabricated nanopore shown in Ⓓ was much smaller than the others, although the same *I*_th_ was used. The cumulative probability of *d*_eff_ confirms that nanopores with *d*_eff_ = 21–26 nm could be fabricated with a yield of approximately 83% (i.e., 15/18). The average *d*_eff_ is 21.7 nm and its standard deviation is 6.41 nm. All observed TEM images of the fabricated nanopores are shown in Supplementary Fig. 4. The effective thickness (*h*_eff_) of the nanopores was estimated by the following equation^53–55^,

7 $${h}_{{\rm{eff}}}=\frac{\pi }{4G}(\sigma {d}_{{\rm{eff}}}^{2}-G{d}_{{\rm{eff}}}),$$

where σ = 0.104 S/cm is the measured conductance of the KCl buffer solution at 21.0 °C. *G* = 150 nS is the conductance of the ionic current through the nanopore, which derives from *I*_th_ = 15 nA at 0.1 V. *h*_eff_ was calculated to be 7.52–16.4 nm for *d*_eff_ = 21–26 nm. According to previous reports^30,53–56^, *h*_eff_ is smaller than the actual membrane thickness. The result in this study is also consistent with this trend. Note that a nanopore was sometimes formed under the top SiN layer (see the TEM image in Supplementary Fig. 5). We subjected 24 membranes to TSB, and the nanopore formation under the top SiN layer was confirmed in 6 membranes. Such cases were excluded from the analysis because it was impossible to precisely measure the area of the nanopore.

**Figure 8:**
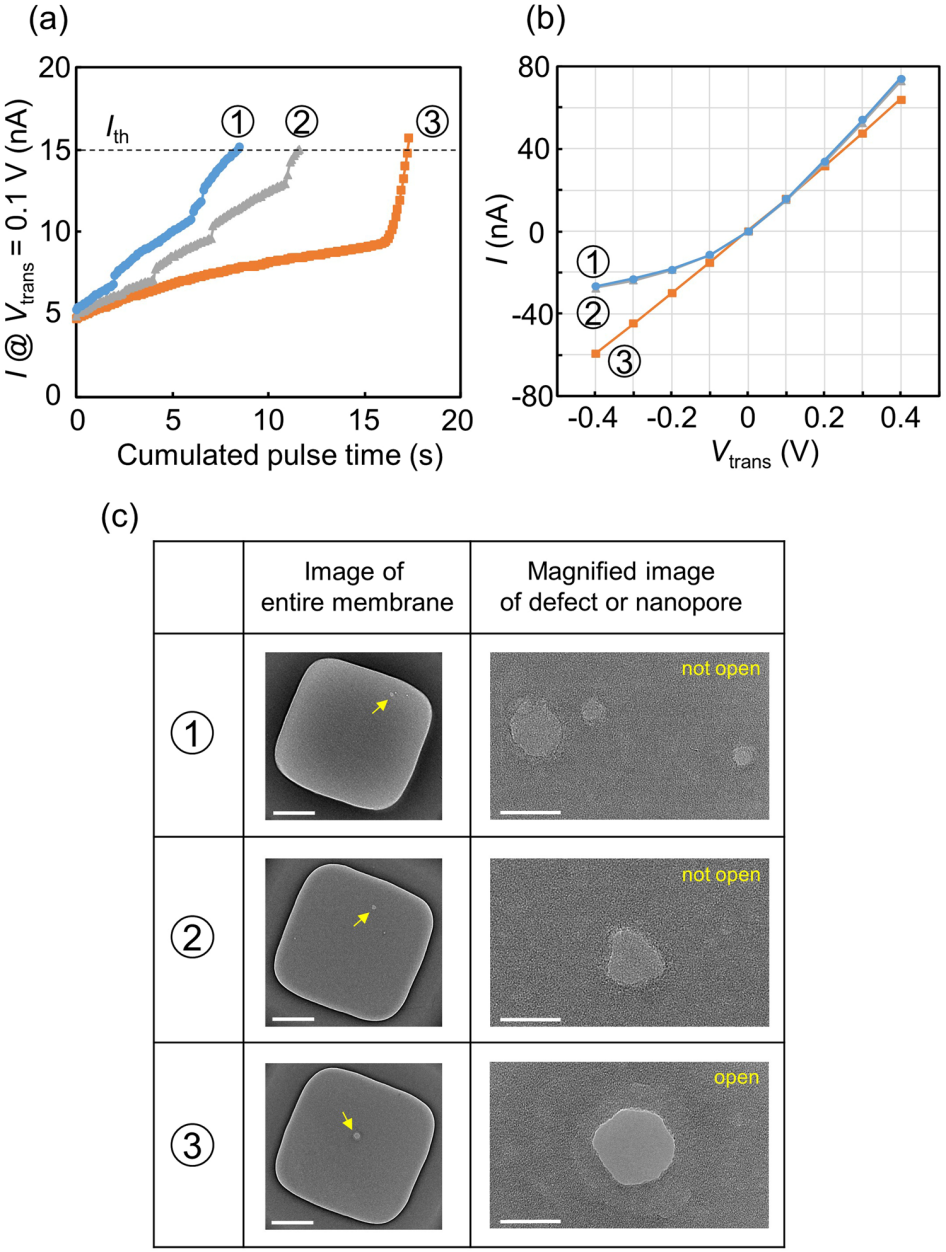
TSB characteristics when the polarity of the voltages in the first and second steps is the same. (**a**) Dependence of the current at *V*_trans_ = 0.1 V and *V*_cis_ = 0 V on the cumulated voltage-pulse time in the second step of TSB. (**b**) *I-V* characteristics across the membranes after the second step of TSB. (**c**) Images of entire membranes after TSB are shown in the left column (scale bars are 200 nm). Yellow arrows indicate local thin portions or a nanopore. Magnified images of the local thin portions or a nanopore are shown in the right column (scale bars are 20 nm).

Figure 8 presents the results of using the same voltage-pulse polarity in the first and second steps of TSB, i.e., *V*_cis_ = 10 V and *V*_trans_ = 0 V with duration of 0.1 s were chosen to generate the pulses. Compared to the results in Fig. 6(b), longer cumulated pulse time (longer than approximately 8 s) was required for the current to reach *I*_th_ (Fig. 8(a)). In addition, rectified *I-V* curves were confirmed (① and ② in Fig. 8(b)) even after the second step of TSB. TEM images of the membranes after TSB are shown in Fig. 8(c). Not nanopores but local thin portions were created in the membranes of ① and ②, and a nanopore was created in the membrane of ③ whose *I-V* curve was ohmic. We tested eight membranes under the same conditions, and nanopore creation was confirmed only in two cases.

The reason why the nanopore fabrication yield differed depending on the polarity of the voltage pulses in the second step is considered as follows. According to reports by Briggs and Kwok *et al*.^28,32^, the major cause of the dielectric breakdown of a SiN membrane is H^+^ incorporation or hole injection. In our experiments, positive and negative charges were thought to be incorporated and fixed in the *cis* and *trans* sides of the local thin portion, respectively, in the first step of TSB. Consequently, in the second step, H^+^ or holes could be injected into the local thin portion more easily when *V*_cis_ < *V*_trans_, leading to fast and high-yield nanopore fabrication.

**Figure 9:**
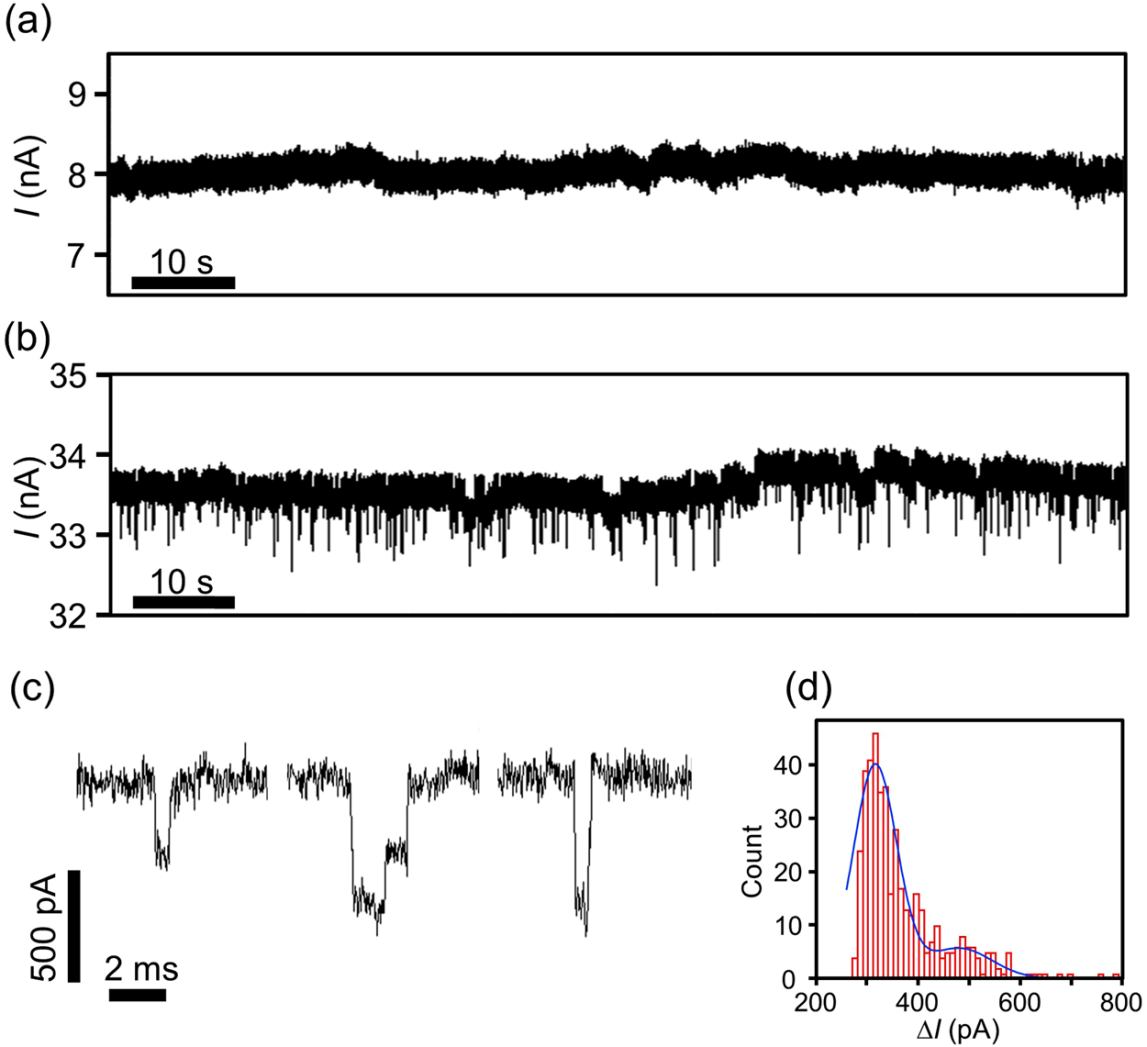
DNA translocation experiments before and after the second step of TSB. Before each measurement, aqueous solution in the *cis* chamber was displaced by a 1 M KCl buffer solution with 10 nM 1-kb double-stranded DNA. Current between the electrodes was monitored at *V*_trans_ = 0.2 V and *V*_cis_ = 0 V. Each data point was low-pass-filtered at 10 kHz. (**a**) A time trace of the current before the second step of TSB. (**b**) A time trace of the current after the second step of TSB. (**c**) Magnified views of typical current-blockade events. (**d**) A histogram of the blockade currents (Δ*I*) occurring during the measurement period of 5 minutes. The histogram is fitted with a double-Gaussian-fit curve.

Finally, the results of the DNA translocation experiments before and after the second step of TSB are shown in Fig. 9. In the TSB, the voltage polarity of the first and second step was opposite (i.e., the TSB conditions were as described in Fig. 6(a)). Prior to the DNA translocation experiment in each stage, the solution in the *cis* chamber was displaced by a 1 M KCl buffer solution with 10 nM 1-kb double-stranded DNA. The current through the membrane was recorded at 0.2 V (i.e., *V*_cis_ = 0 V and *V*_trans_ = 0.2 V). The time trace of the current before the second step is shown in Fig. 9(a). No current-blockade events (i.e., DNA translocation events) were confirmed, as we expected. This result also strongly supports the absence of nanopores in the local thin portion. Of course, DNA translocation events were clearly confirmed after the second step (Fig. 9(b)). Magnified views of three typical current-blockade events are shown in Fig. 9(c). These current patterns are well known as results of unfolded and folded DNA passing through a nanopore^25,27,28,32,46,57–59^. Figure 9(d) presents a histogram of the current-blockade values (Δ*I*). Δ*I* was defined as the mean current-blockade value of one current-blockade event. The histogram was fitted with a double-Gaussian curve with two peaks, which correspond to the unfolded and folded DNA translocation events.

The results of the DNA translocation experiments after CBD with *I*_c_ = 6.5 μA and 10 μA are presented in Supplementary Fig. 6. Although current-blockade spikes were observed in each case, the fluctuation of the baseline current was larger than the current-blockade values. This baseline instability is thought to be due to the trapping and detrapping of carriers at the local thin portion. As shown in Fig. 4, the area of the local thin portion became larger as *I*_c_ increased, leading to larger fluctuation in the baseline current.

## Discussion

The stable fabrication of nanopores in 20-nm-thick SiN membranes via dielectric breakdown was examined. First, we found that it was practically impossible to fabricate a single nanopore in the membrane via the conventional CBD approach. Not a nanopore but a local thin portion was formed in the membrane when the applied voltage was 20 V and the cut-off current (*I*_c_) was less than 3 μA. On the other hand, multiple nanopores were formed in the membrane when *I*_c_ was greater than 3 μA. The local thin portion had electrical conductivity, but did not allow DNA translocation. The current through the local thin portion exhibited a rectified conductance, which was attributed to the fixed charges trapped in the local thin portion during CBD.

The generation of multiple nanopores in the case of *I*_c_ > 3 μA occurred within a very short time (less than 0.2 s). Therefore, this generation of multiple nanopores could not be interpreted as an aggregation of independent weakest-link events. We cannot currently provide a credible explanation for this phenomenon. The propagation of Joule heating energy by phonons in the membrane and imbalanced local stress in the membrane are thought to be possible causes.

To stably fabricate a single nanopore in a membrane under such circumstances, we proposed the technique called “two-step breakdown” (TSB). In the first step of TSB, a local thin portion was formed in the membrane by CBD with an applied voltage of 20 V and *I*_c_ of 1 μA. Then, the created local thin portion was penetrated by voltage pulses (i.e., 10 V pulses with durations of 0.1 s). The polarity of the voltage used in each step is an important factor. A single nanopore with an effective diameter of 21–26 nm could be successfully fabricated with a high yield of 83% when the polarity of the pulse voltages in the second step was opposite to that of the voltage during CBD in the first step. On the other hand, a nanopore could be fabricated with a probability of only 25% when voltages with the same polarity were used in the first and second steps.

TSB is a simple and quite inexpensive technique that enables the fabrication of solid-state nanopores up to approximately 25 nm in diameter. Consequently, we believe TSB will accelerate the research and development of large-molecule detection with nanopores. In addition, this study provides important knowledge related to semiconductor reliability physics. In particular, the step-by-step visualization of the dielectric breakdown presented in this study has been always the subject of interest in research on the dielectric breakdown of field-effect transistor (FET) gate insulators.

## Methods

### Fabrication of membranes

The SiN membranes were fabricated on an 8-inch silicon wafer with a thickness of 725 μm. First, a SiN layer with a thickness of 20 nm was deposited on both sides of the Si wafer via low-pressure chemical vapour deposition (reacting gases: SiH_2_Cl_2_-NH_3_; flow ratio: SiH_2_Cl_2_:NH_3_ = 1:25; 770 °C). Then, a SiO_2_ sacrificial layer with a thickness of approximately 260 nm was deposited on the SiN layer on the front side of the wafer, and a SiN layer with a thickness of approximately 90 nm was deposited on both sides of the wafer. Then, the top SiN layer in each square area approximately 600 × 600 nm^2^ in size and the backside SiN layer in each corresponding 1038 × 1038 μm^2^ square area were subsequently etched by reactive-ion etching, followed by etching of the Si substrate with tetramethylammonium hydroxide (TMAH) at 85 °C for 9 hours. After etching of the Si substrate, the wafer was diced into chips. Finally, the SiO_2_ sacrificial layer was etched with potassium hydroxide (33 wt.% solution of KOH for 25 min at 75 °C) before the dielectric-breakdown experiment of the SiN membrane.

### Analyses of the SiN membrane by TEM and XPS

Observations of the SiN membranes were performed using a field-emission transmission electron microscope (JEM-2100F (HRP), 200 kV, JEOL, Ltd.). Before the observations, the membranes were immersed in deionized water for a day to remove any salt residues. The areas of the fabricated nanopores were measured using image processing software (ImageJ, National Institutes of Health, Bethesda, MD, USA).

Cross-sectional images of the SiN layers were captured using a scanning transmission electron microscope (HD 2700, 200 kV, Hitachi High-Technologies Corp.). An investigation of the composition ratio of the SiN film was performed by XPS (PHI 5000 VersaProbe II, X-ray: Al Kα, ULVAC-PHI, Inc.).

### Setup for dielectric-breakdown experiments

Initially, the SiN membrane was mounted onto a custom-built acrylic flow cell. Separated by the membrane, two chambers (each with a volume of 90 μL) were formed in the flow cell: a *cis* chamber and a *trans* chamber. Both chambers were filled with a buffer solution consisting of 1 M potassium chloride, 10 mM Tris-HCl, and 1 mM EDTA buffer at pH 7.5. Two Ag/AgCl electrodes were immersed in both solutions to ensure electrical contact.

Application of the constant voltage, measurement of the current through the membrane during CBD, and measurement of the *I-V* characteristics were performed using a 4156B Precision Semiconductor Parameter Analyzer (Agilent Technologies, Inc.). The cut-off current for CBD was set as the current compliance value of the 4156B system. The pulse voltages used in the second step of TSB were applied with a 41501B SMU and Pulse Generator Expander (Agilent Technologies, Inc.).

### Setup for measuring DNA translocation through a nanopore

Prior to the measurements of DNA translocation through a nanopore, the aqueous solution in the *cis* chamber was displaced by a 1 M KCl buffer solution with 10 nM 1-kb double-stranded DNA (NoLimits, Fermentas, Burlington, Ontario, Canada). The ionic-current measurements shown in Fig. 9 were performed using a patch-clamp amplifier (Axopatch 200B, Axon Instruments, Union City, CA). The detected current was low-pass-filtered with a cut-off frequency of 10 kHz using a four-pole Bessel filter and then digitized with an NI USB-6281 18-bit DAQ AD converter (National Instruments, Austin, TX) at 50 kHz. Finally, the current was recorded on the hard disk of a personal computer. Current-blockade events were identified and analysed by Clampfit 10.2 software (Molecular Devices). All the measurements described above were performed at room temperature.

## Electronic supplementary material

Supplementary Information
