# Supplementary material for: Two-step breakdown of a SiN membrane for nanopore fabrication: Formation of thin portion and penetration
Source: Sci Rep. 2018 Jul 4;8:10129. doi: 10.1038/s41598-018-28524-5 (PMC6031669; doi:10.1038/s41598-018-28524-5)
Supplement: Supplementary file 1 — Supplementary Information [file 41598_2018_28524_MOESM1_ESM.doc]

**Supplementary Information for**

**Two-step breakdown of a SiN membrane for nanopore fabrication: Formation of thin portion and penetration**

Itaru Yanagi*, Hirotaka Hamamura, Rena Akahori and Ken-ichi Takeda

Hitachi Ltd., Research & Development Group, Center for Technology Innovation - Healthcare, 1-280, Higashi-koigakubo, Kokubunji, Tokyo, 185-8603, Japan

**Supplementary Figure 1. Cross-sectional transmission electron microscopy (TEM) images of the SiN/SiO_2_/SiN multilayers.** The thickness of the bottom SiN layer, middle SiO_2_ layer and top SiN layer is approximately 20 nm, 260 nm and 90 nm, respectively. Scale bars are 30 nm.


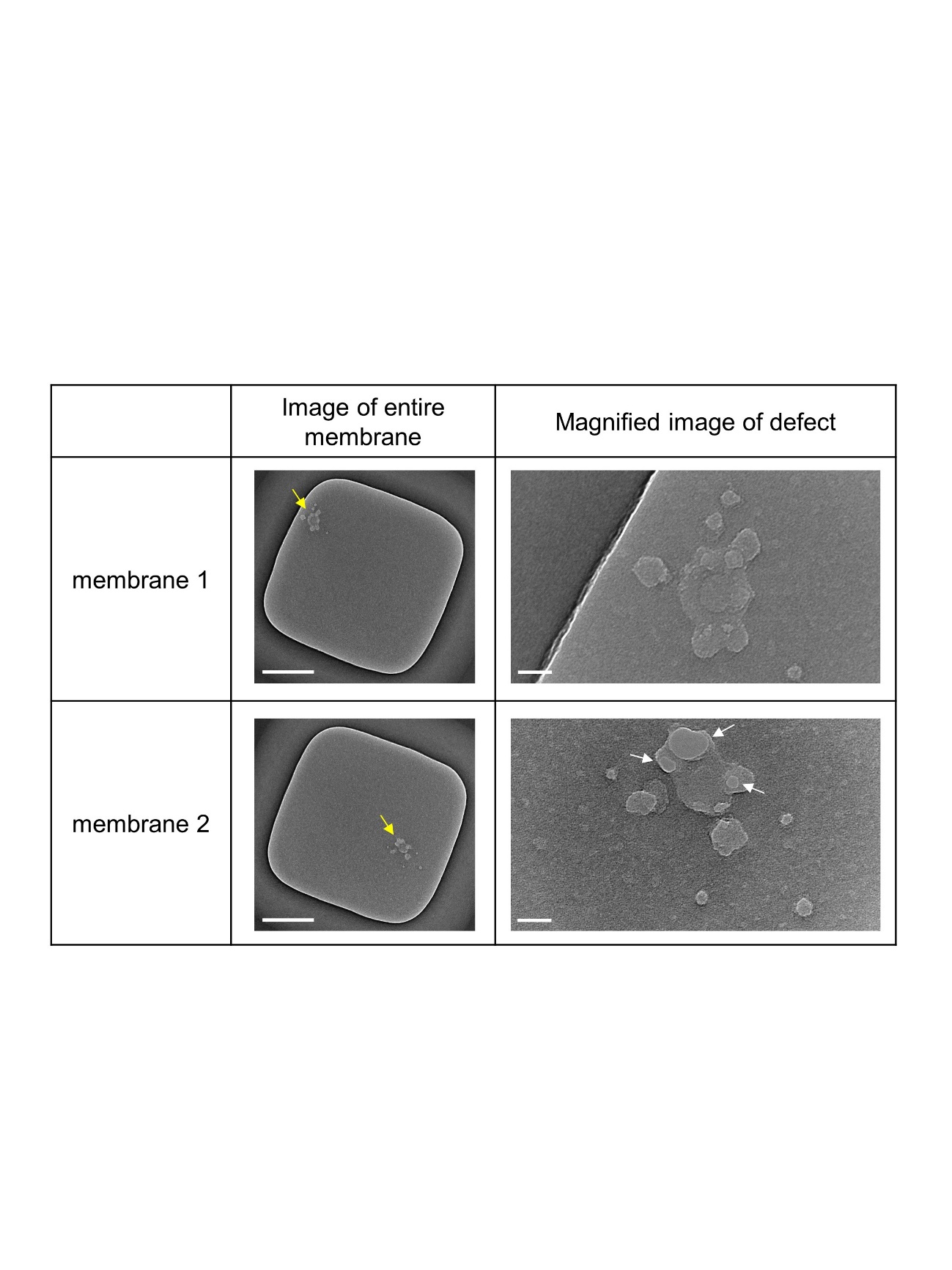


**Supplementary Figure 2. TEM images of the membranes after CBD with *I*_c_ = 4.75 μA.** Two membranes were tested under the same conditions. Images of entire membranes are presented in the left column (scale bars are 200 nm). Defective portions are indicated by yellow arrows. Magnified images of the defective portions are presented in the right column (scale bars are 20 nm). From the magnified image of the membrane 1, only local thin portions were confirmed (i.e., no nanopores were confirmed). On the other hand, multiple nanopores (indicated by white arrows) were confirmed in the membrane 2. This result means that the control of the number of nanopores is practically impossible by tuning *I*_c_.


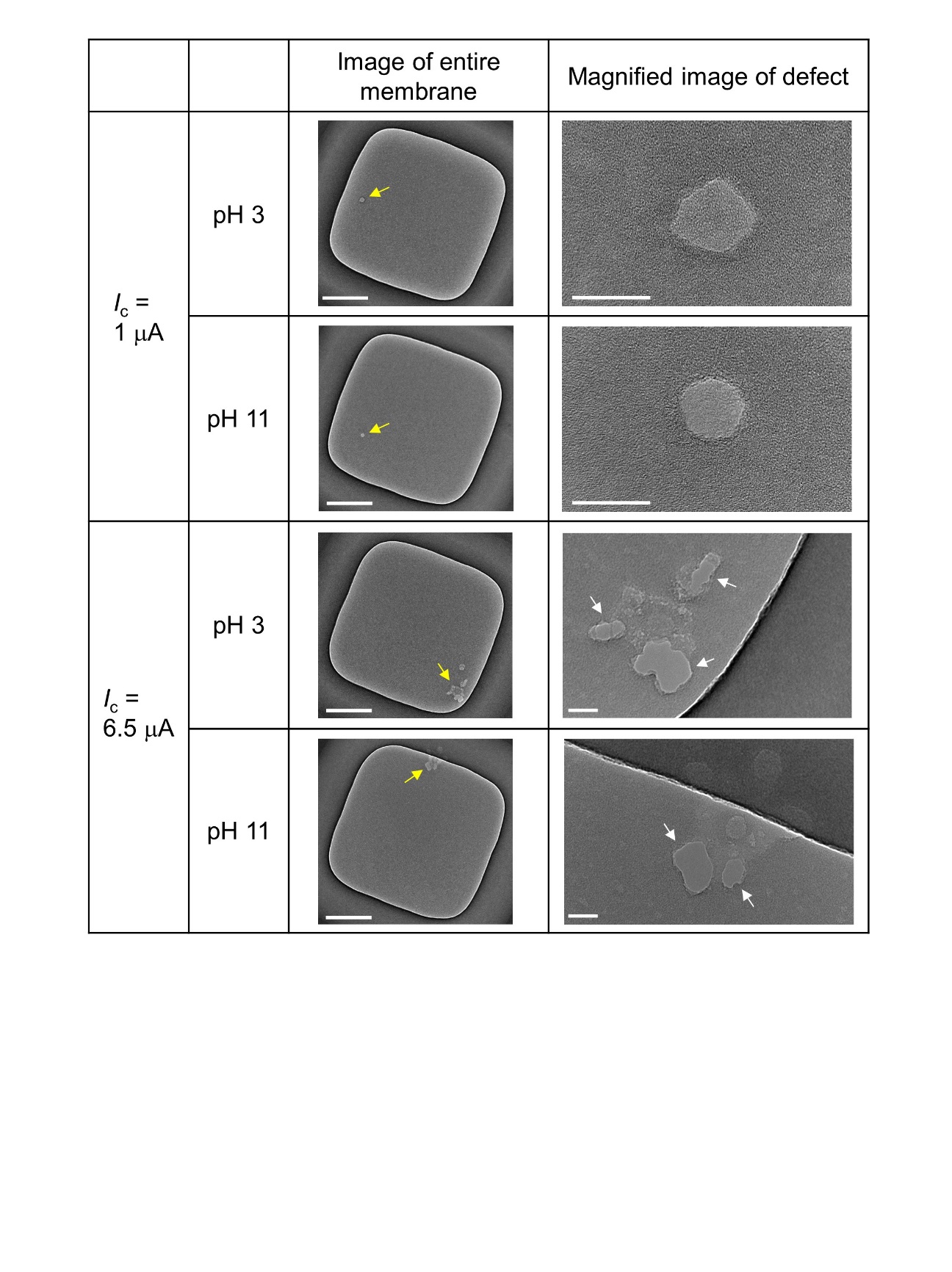


**Supplementary Figure 3. TEM images of the membranes after CBD with different cut-off currents (*I*_c_) and pH conditions.** Images of entire membranes are presented in the left column (scale bars are 200 nm). Defective portions are indicated by yellow arrows. Magnified images of the defective portions are presented in the right column (scale bars are 20 nm). The pH of KCl aqueous solutions was adjusted to 3 or 11 using small amounts of HCl or KOH. Only a local thin portion was created in each of pH conditions when *I*_c_ = 1 μA. On the other hand, multiple nanopores (indicated by white arrows) were generated in each of pH conditions when *I*_c_ = 6.5 μA. This tendency is the same as that in the case of pH 7.5.


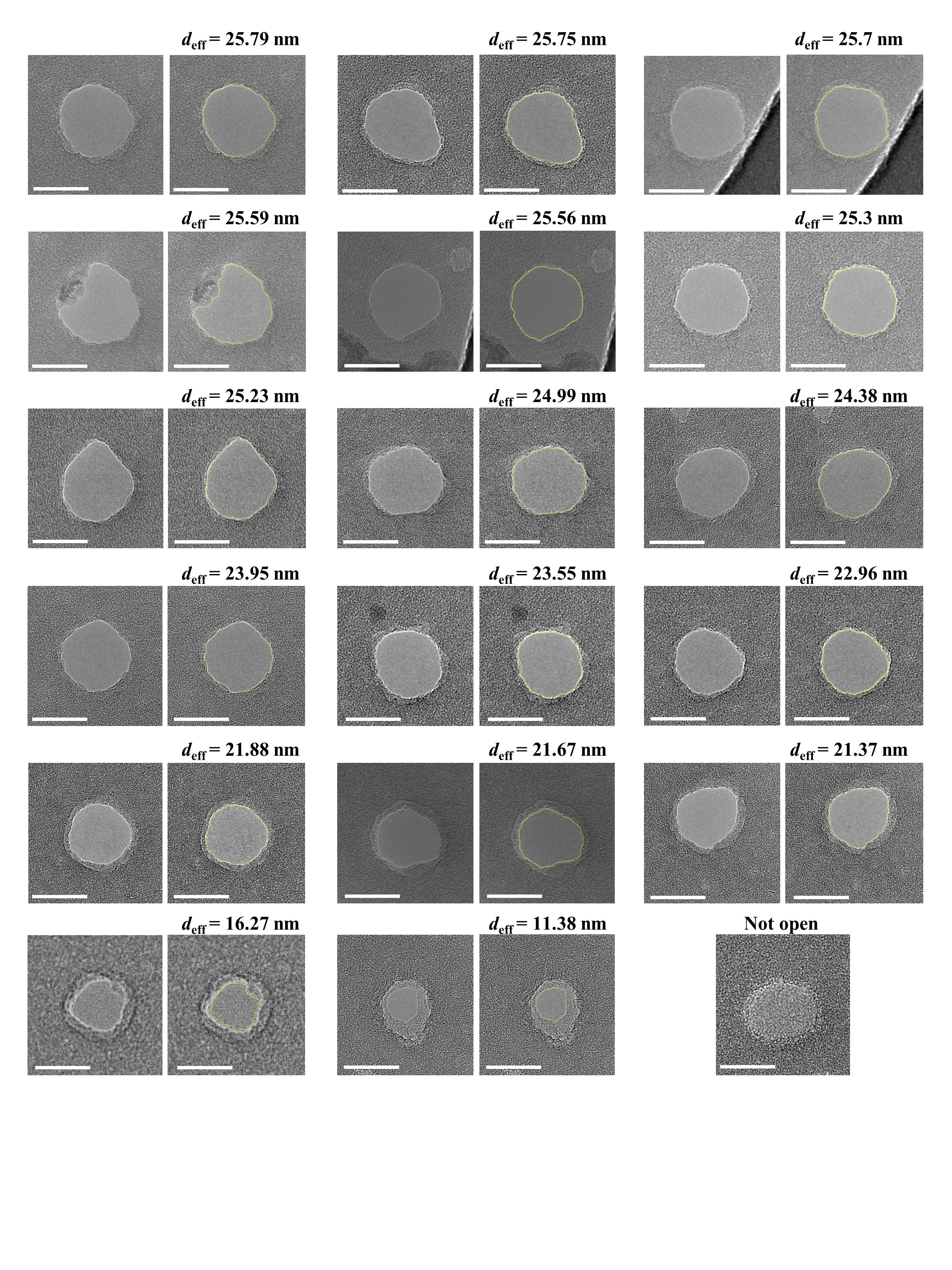


**Supplementary Figure 4. Magnified images of all (N=18) observed nanopores fabricated by two-step breakdown (TSB) when the polarity of the voltage pulses in the second step was set opposite to the polarity of the voltage in the first step.** Scale bars are 20 nm. Each left image shows the raw version of each right image. In each right image, the area of the nanopore is surrounded by a yellow line and was measured using image processing software (ImageJ). Each effective nanopore diameter was calculated by substituting the measured area into equation (6).

**Supplementary Figure 5. A nanopore fabricated under the top SiN layer.** The scale bar is 200 nm. The yellow arrow indicates the fabricated nanopore under the top SiN layer. Such cases were excluded from the effective diameter analysis.

**Supplementary Figure 6. DNA translocation experiments after CBD.** Before each measurement, the aqueous solution in the *cis* chamber was displaced by a 1 M KCl buffer solution with 10 nM 1-kb double-stranded DNA. Each data point was low-pass-filtered at 10 kHz. (a) A time trace of the current after CBD with *I*_c_ = 6.5 μA. The current was monitored at *V*_trans_ = 0.2 V and *V*_cis_ = 0 V. (b) A time trace of the current after CBD with *I*_c_ = 10 μA. The current was monitored at *V*_trans_ = 0.13 V and *V*_cis_ = 0 V.
